# Consumption of *Lactobacillus casei* Fermented Milk Prevents *Salmonella* Reactive Arthritis by Modulating IL-23/IL-17 Expression

**DOI:** 10.1371/journal.pone.0082588

**Published:** 2013-12-10

**Authors:** Mariángeles Noto Llana, Sebastián Hernán Sarnacki, María del Rosario Aya Castañeda, María Isabel Bernal, Mónica Nancy Giacomodonato, María Cristina Cerquetti

**Affiliations:** Instituto de Microbiología y Parasitología Médica (IMPaM-CONICET) and Departamento de Microbiología, Parasitología e Inmunología, Facultad de Medicina, Universidad de Buenos Aires, Buenos Aires, Argentina; Virginia Tech, United States of America

## Abstract

Reactive arthritis is the development of sterile joint inflammation as a sequel to a remote infection, often in the gut. We have previously shown that a low dose of *S*. enteritidis inoculated to streptomycin-pretreated mice generates a self-limiting enterocolitis suitable for studying reactive arthritis. Here we show that consumption of *Lactobacillus casei* prior to infection abolishes intestinal and joint inflammation triggered by *Salmonella*. BALB/c mice were sacrificed after infection; intestinal and joint samples were analyzed for histological changes and expression of cytokines. TNF-α was measured by ELISA and the expression of IL-1β, IL-6, IL-10, IL-17, IL-23 and TGF-β was assessed by qPCR. *L. casei* consumption prevented *Salmonella*-induced synovitis, the increment of TNF-α in knees and the increase of IL-17 expression in popliteal and inguinal lymph nodes. At intestinal level consumption of *L. casei* drastically diminished *S*. enteritidis invasiveness and shortened splenic persistence of the pathogen. Bacterial loads recovered at days 2 and 5 from Peyer’s patches were 10-fold lower in mice fed with *L. casei*. In accordance, we found that the augment in gut permeability induced during enterocolitis was decreased in those animals. Consumption of *L. casei* prior to infection failed to increase anti- inflammatory molecules such as IL-10 and TGF-β in the intestine. On the other hand, consumption of *L. casei* abrogated the expression of TNF-α, IL-17, IL-23, IL-1β and IL-6 in cecum and mesenteric lymph nodes. These cytokines are needed for differentiation of immune cells involved in the development of reactive arthritis such as Th17 and γδ T cells. Trafficking of these inflammatory cells from the gut to the joints has been proposed as a mechanism of generation of reactive arthritis. Our results suggest that *L. casei* consumption prevents *Salmonella-*induced synovitis by altering the intestinal milieu necessary for differentiation of cells involved in the generation of joint inflammation.

## Introduction

A close association exists between the generation of gut inflammation and joint disease. An early clue to this link was the observation that certain bacterial enteropathogens can induce reactive arthritis (ReA). In this sense, ReA is defined as the sterile inflammation of joint tissues following a distant mucosal infection, often gastrointestinal [1]. In developing countries one-third of all ReA cases are triggered by *Salmonella* enterocolitis [2].

*Salmonella enterica* serotype Typhimurium (*S*. Typhimurium) and *S*. Enteritidis, the two most abundant serotypes for salmonellosis, elicit an acute inflammatory response in the intestinal mucosa of humans that can be modeled using streptomycin-pretreated mice [3]. This inflammatory reaction is initiated by direct contact of *Salmonella* with host cells, such as epithelial cells, macrophages or dendritic cells, followed by an amplification of inflammatory responses in tissue [4]. As a result, changes in gene expression are observed in the intestinal mucosa during *Salmonella* infection, including markedly increased mRNA levels of interleukin-17 and IL-23 genes [5]. T cells play an important role in amplifying inflammatory responses in the cecal mucosa; trafficking of these gut inflammatory cells towards the joints has been proposed as a mechanism of generation of ReA [6].

Probiotics are defined by FAO and WHO as live microorganisms which when administered in adequate amounts, confer a health benefit to the host [7]. Orally administered probiotics exhibit widespread effects on gut homeostasis and immunomodulation of both mucosal and systemic immunity. It is well documented that probiotics may counterweight aggressive enteric pathogens in the gut, reinforce the barrier function of the epithelium and contribute in the regulation of innate and adaptive immune responses of the host under healthy or pathogenic conditions [8]. *Lactobacillus casei* DN-114 001 is a probiotic strain that survives intestinal transit [9] and exerts beneficial effects in vivo. This probiotic strain is able to modify the digestive microflora and enhance the immune system during its transit in the digestive tract [10,11]. It was also shown that *Lactobacillus casei* DN-114 001 is able to reduce the incidence and duration of diarrhea in children [12,13]. Moreover, this probiotic interacts with human intestinal mucosa and can markedly reduce the mucosal release of inflammatory cytokines in active Crohn’s disease [14,15]. Nevertheless, the use of probiotics to prevent ReA has not been investigated, probably because of the lack of an adequate experimental model. In this regard, investigation on the pathogenesis of ReA is difficult because of the limited studies that can be performed in humans; therefore, the availability of animal models is crucial. We have recently showed that a low dose of *S*. Enteritidis inoculated to streptomycin-pretreated mice generates a self-limiting enterocolitis model useful for studying ReA [16]. In the present work, using this animal model of intestinal infection we were able to determine the protective role of *Lactobacillus casei* DN-114 001 fermented milk (LCFM) on the development of ReA associated to *Salmonella* enterocolitis.

## Materials and Methods

### Mice

Six to 8 – week old female BALB/c mice were obtained from our vivarium, maintained under standard conditions and provided with food and water *ad libitum*. At the end of each experiment, mice were killed with carbon dioxide. All experimental protocols were approved by the Animal Ethics Committee, University of Buenos Aires.

### Bacteria

Wild-type strain of *Salmonella enterica* serovar Enteritidis #5694 (*S*. Enteritidis) was used to infect mice. Bacteria were cultured in trypticase soy broth at 37 °C, 200 cycles per minute, then pelleted by centrifugation and suspended to the appropriate density in saline solution. In all cases the number of bacteria was determined by plating appropriate dilutions on trypticase soy agar plates. 

### *Salmonella* infection and generation of enterocolitis

Mice were pretreated with 20 mg of streptomycin (Sigma Aldrich) given intragastrically [17] and 24 h later they received 3-4 x 10^3^ CFU of *S*. Enteritidis by the same route. For intragastric infection, 0.2 ml of the bacterial suspension was introduced into the stomach with a 21 G blunt needle on a 1.0 ml plastic syringe.

### Probiotic administration

Commercially-available fermented milk containing *Lactobacillus casei* DN-114001 (LCFM, Danone S.A.) was used in this study. LCFM was given to mice *ad libitum*, as described elsewhere [18] during 7 consecutive days prior to *S*. Enteritidis infection. Mice ingested an average dose of 1 x 10^8^ CFU of LCFM per day.

### Experimental groups

Throughout this study five different experimental groups where used, as shown in Table 1. Results obtained from Strep group were similar to those presented by the Control and *L. casei* + Strep group; therefore, they were not described in Result section unless otherwise stated. Samples were taken at days 2, 5 and 14 post *Salmonella* infection.

**Table 1:** Experimental groups used throughout this study.

**Group of mice**	**Probiotic**	**Streptomycin**	***S*. Enteritidis**
**EC**	**-**	**+**	**+**
***L. casei* + EC**	**+**	**+**	**+**
**Control**	**-**	**-**	**-**
***L. casei* + Strep**	**+**	**+**	**-**
**Strep**	**-**	**+**	**-**

### Bacterial colonization and persistence

At the indicated times post infection mice were sacrificed and bacterial loads were analyzed. All Peyer´s patches located along the large intestine (6 to 8) and one third of the spleen were removed aseptically from each animal and homogenized in sterile saline solution. Samples were diluted appropriately in saline and plated on *Salmonella*- *Shigella* (SS) agar. Samples were also cultured for 18 h in selenite broth for enrichment. *Salmonella*-like colonies appearing on SS plates were grown on triple-sugar-iron agar slants and tested for somatic antigen O9. 

Persistence and colonization of bacteria in knee and popliteal and inguinal lymph nodes were also studied by PCR. Tissues were homogenized in 1% PBW (Peptone Buffered Water). After 24 h of enrichment at 37° C, DNA extraction was performed using the phenol-chloroform technique [19]. A standard PCR of 45 cycles was carried out using selective primers to amplify *invA* and *sopA* genes, of 285 pb and 113 pb products respectively. Primers used were: forward 5´-CTGAAATTATCGCCACGTTCGGGCAA – and reverse 5´-CATCGCACCGTCAAAGGAACC -3´ to amplify *invA* gene [19] and forward 5´-TCCACCGTGAAGTTGATTG -3´ and reverse 3´-GCACTGAGGATGTGCTGGTA –5´ [20] for *sopA* gene. The cycling programme was 95° C for 10 s, 55 ° C for 10 s and 72° C for 15 s and one cycle of 40° C for 30 s. Twenty µl aliquots of the reaction mixtures were electrophoresed through 2.0% agarose gel and fragments were revealed by staining with ethidium bromide. 

### Histological analysis

Intestinal samples were fixed in formalin and processed by standard procedures for paraffin embedding. Knee joints were dissected, fixed in formalin for 2 days, decalcified in EDTA for 30 to 40 days, and then embedded in paraffin. Standard sections of 5 µm were prepared and stained with haematoxylin-eosin (HE) using routine histology techniques. An experienced pathologist blinded to the experimental protocol evaluated findings of intestine and joint abnormalities. Synovial alterations were scored as 0 = no changes; 1 = slight thickening of synovial cell layer (up to 3 layers of synoviocytes) accompanied by congestion and oedema of the external membrane; 2 = moderate thickening of synovial cell layer (3 to 5 layers of synoviocytes) accompanied by congestion and oedema of the external membrane; 3 = severe thickening of synovial lining (more than 5 layers) accompanied by congestion and oedema of the external membrane. Intestinal alterations were scored as 0 = no changes; 1 = oedema and/or villous capillary dilatation with epithelial preservation; 2 = focal mucosa irritation characterized by villous hydrops and capillary dilatation with mild to moderate mononuclear cell infiltration; 3 = similar to 2 but of extended distribution; 4 = mucosal alteration with mononuclear cell infiltration and loss of epithelial integrity.

### Quantitative real-time reverse transcriptase-polymerase chain reaction

Total RNA was extracted from tissues using Trizol reagent (Life Technologies, Inc, Carlsbad, CA) at different time points according to the experiment. Total RNA (1 µg per sample) was reverse transcribed with oligo(dT) as primer using Expand Reverse Transcriptase (Promega Corporation, Madison, WI) according to the manufacturer’s protocol. Quantitative real-time reverse transcriptase-polymerase chain reaction was performed using SyBr Green PCR kit (Applied Biosystems Inc, Foster City, CA) in an Applied Biosystems 7500 sequence detector. Primer sequences are described in Table 2. All samples were analyzed in the same run for 18s expression for normalization. Polymerase chain reaction parameters were 50° C for 2 min, 94° C for 2 min, and 40 cycles of 94° C for 30 s and 60° C. Quantification of gene expression was calculated using the comparative threshold cycle (Ct) method, normalized to the 18s control and efficiency of the RT reaction (relative quantity, 2^-∆∆Ct^). The replicates were then averaged, and fold induction was determined, considering the value in “Control” group as 1 [21]. 

**Table 2:** Oligonucleotides primers used for quantitative real-time reverse transcriptase-PCR assays.

**mRNA Targeted**	**Sequence** (5’→3’) **^*a*^**	**Accession numbre**	**PCR product size (nt)**	**Ref.**
TNF-α	ATGAGCACAGAAAGCATGATC (F)	U68415	276	[21]
	TACAGGCTTGTCACTCGAATT (R)			
IL-17	GCTCCAGAAGGCCCTCAGA (F)	U43088	142	[5]
	AGCTTTCCCTCCGCATTGA (R)			
IL-1β	TTGACAGTGATGAGAATGACC (F)	NM_008361	252	[21]
	CAAAGATGAAGGAAAAGAAGG (R)			
IL-6	TGATGCACTTGCAGAAAACAA (F)	NM_031168	328	[21]
	GGTCTTGGTCCTTAGCCACTC (R)			
IL-23	TGTGCCTAGGAGTAGCAGTCCTGA (F)	NM_031252	226	[5]
	TTGGCGGATCCTTTGCAAGCAGAA (R)			
IL-10	CCAAGCCTTATCGGAAATGA (F)	NM_010548	162	[21]
	TTTTCACAGGGGAGAAATCG (R)			
TGF-β	ACCAACTACTGCTTCAGCTC (F)	NM_011577	194	[22]
	TGTTGGTTGTAGAGGGCAAG (R)			
18s rRNA	AACACGGGAAACCTCACCC(F)	GU372691	103	[21]
	CCACCAACTAAGAACGGCCA (R)			

Primers were purchased from Invitrogen Inc. and were designed according to the DNA sequence information available for *Mus musculus* (*M. musculus* blast server BLAST Server Database at www.sanger.ac.uk).

^a^ F, forward primer; R, reverse primer.

### Cytokine Analysis

For TNF-α determination, knee samples were obtained 5 days after oral inoculation with 3-4 x10^3^ CFU of *S*. Enteritidis from mice of EC and *L. casei* + EC groups. Tissue homogenates from 5 animals were pooled and subjected to centrifugation (12.000 rpm, 1 min) to pellet all cell debris prior to concentration using an Amicon Ultra-4 Centrifugal Filter Unit (Merck Millipore). Supernatants were stored at -20°C until further use. Analyses were conducted using commercially available enzyme-linked immunosorbent assay (ELISA) kits (R&D Systems, Minneapolis, MN) according to manufacturer's instructions. Cytokine levels were expressed as picogram per ml (pg/ml).

### Intestinal permeability in vivo

The intestinal permeability was measured by determining the amount of FITC- dextran in blood after it was orally administered as described previously [23]. Briefly, each mouse received 440 mg/kg of body weight of FITC- dextran (molecular weight 4.4 kDa; Sigma-Aldrich) by gavage. A blood sample, obtained 5 h later, was first centrifuged (3.000 rpm at 4° C) for 30 min, serum was collected and added to a 96- well microplate. The concentration of FITC- dextran was determined by spectrophotofluorometry with an excitation wavelength of 483 nm and an emission wavelength of 525 nm using serially diluted samples of the marker as standard.

### Statistical analysis

Statistically significant differences between experimental groups were determined by one-way analysis of variance (ANOVA) followed by Tukey test for multiple comparisons. Statistical analysis was performed using the software program Prism 4.0 (GraphPad Software, San Diego, CA, USA). P-values less than 0.05 were considered statistically significant.

## Results

### Consumption of LCFM prevents joint inflammation associated with *Salmonella* enterocolitis

**Figure 1:**
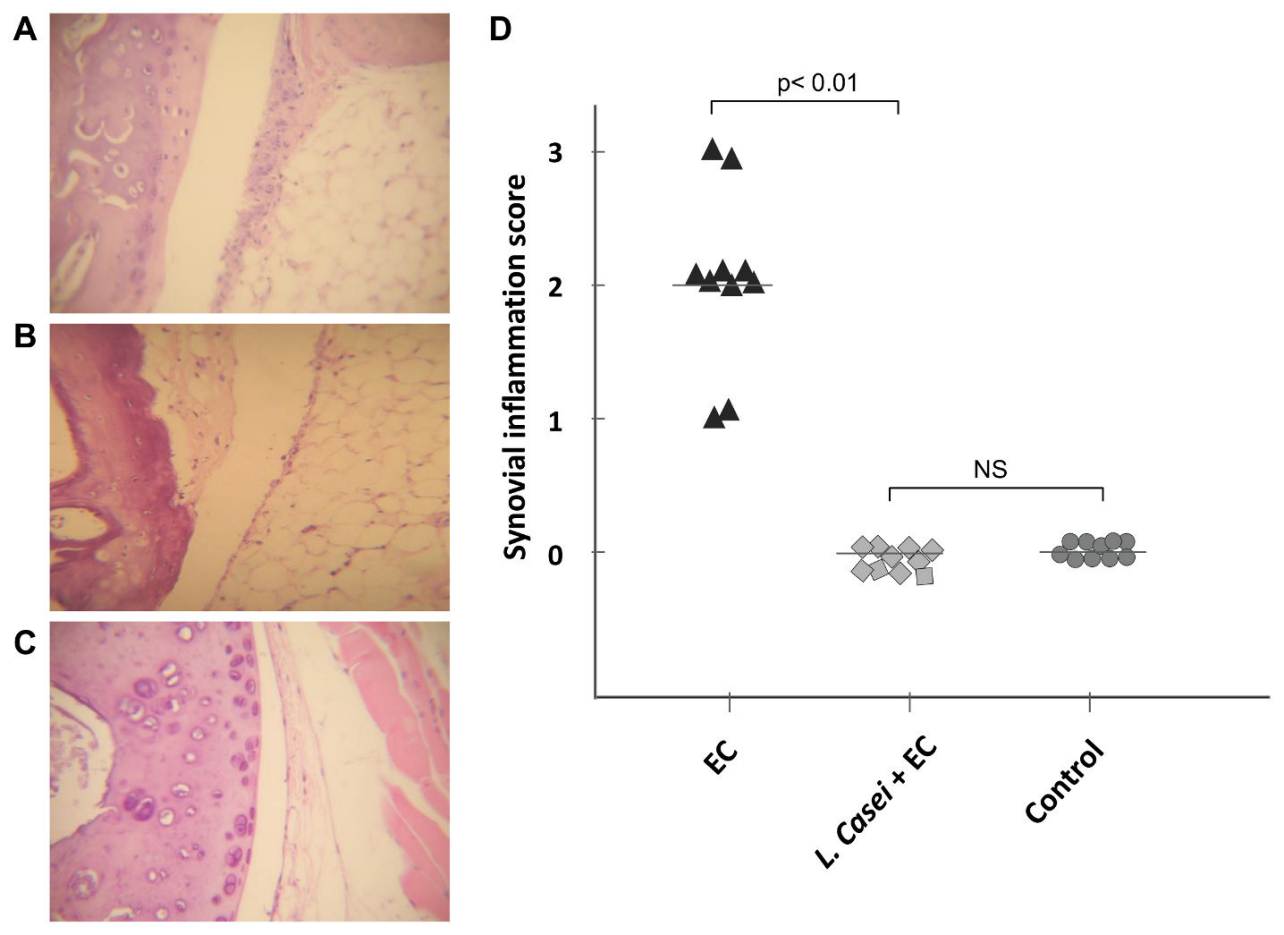
Effect of LCFM consumption on joint pathology during *Salmonella* enterocolitis. A to C: Histology of knee joints 5 days after oral inoculation with the pathogen. D: Synovial inflammation scores. EC group: mice received 20 mg of streptomycin 24 h before intragastric infection with 3-4 x 10^3^ CFU of *S*. Enteritidis. *L. casei* + EC group: mice received the probiotic for a week before enterocolitis onset. Control group: untreated mice. (A) EC group: moderate hyperplasia, with 3 to 5 layers of synoviocytes (arrows). (B) *L. casei* + EC group: normal synovial capsule (arrows), undistinguishable from Control group. (C) Control animals: normal synovial capsule (arrows). HE; 40x. Data were collected from 3 independent experiments.

Experiments were conducted using a model in which streptomycin-pretreated mice are infected with a low dose of *S*. Enteritidis in order to induce enterocolitis [16]. Mice received 20 mg of streptomycin 24 h before intragastric infection with 3 to 4 x 10^3^ CFU of the pathogen (EC group). The experimental group was fed with LCFM for one week prior to the induction of *Salmonella* enterocolitis (*L. casei* + EC group). Mice treated with streptomycin only (Strep group), fed with *L. casei* and streptomycin (*L. casei* + Strep) and animals without any treatment (Control group) were included as controls. As shown earlier [16], 5 days after the induction of *Salmonella* enterocolitis, animals of the EC group presented synovitis with moderate hyperplasia of the synovial membrane and mononuclear infiltration (Figure 1A); lesions observed corresponded to a pathological score of 2 (Figure 1D). Interestingly, mice fed with the LCFM one week prior to the onset of enterocolitis (*L.casei* + EC) did not present any joint alteration (Figure 1B); their synovial membranes were healthy and similar to those from the Control group (Figure 1C) with a pathological score of 0 (Figure 1D). Results obtained from *L. casei* + Strep group were similar to those presented by the Control group (data not shown).

**Figure 2:**
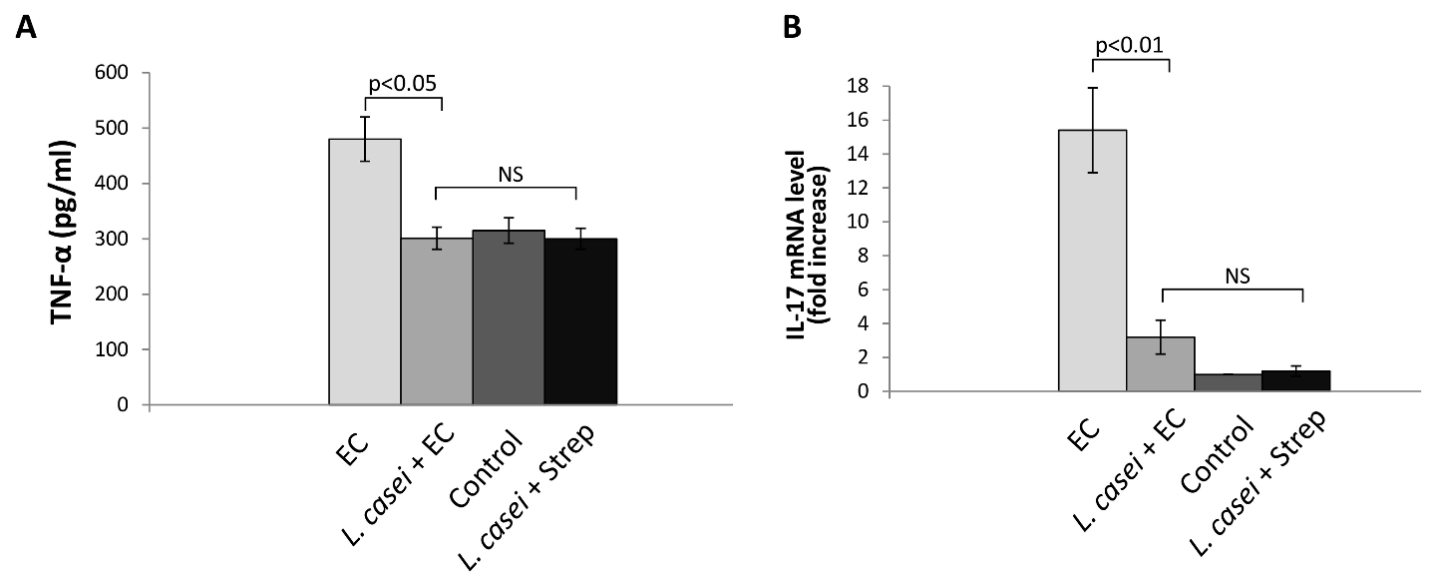
Effect of LCFM consumption on joint during *Salmonella* enterocolitis. **A**. TNF-α was measured in joint homogenates by ELISA 5 days after oral infection with the pathogen. Five animals per group were analyzed. **B**. IL-17 expression was analyzed by qPCR at day 5 post infection in joint draining lymph nodes. Seven animals per group were analyzed. EC group: mice received 20 mg of streptomycin 24 h before intragastric infection with 3-4 x 10^3^ CFU of *S*. Enteritidis. *L. casei* + EC group: mice received the probiotic for a week before enterocolitis onset. Control group: untreated mice. *L. casei* + Strep group: animals were fed with the probiotic for a week and then received streptomycin. Results are expressed as mean +/- SD. (NS): no significant differences. Data were collected from 3 independent experiments.

Because local production of TNF-α appears to be responsible for synovial inflammation induced during *S*. Enteritidis intestinal infection [16], we analyzed whether consumption of the probiotic modifies the production of this inflammatory molecule. The amount of TNF-α was determined by ELISA in joint homogenates 5 days after the onset of *S*. Enteritidis enterocolitis. As shown in Figure 2A, the increase of articular TNF-α observed in animals suffering enterocolitis (EC group) was abrogated by the consumption of LCFM prior to *S*. Enteritidis infection (*L. casei* + EC group). The amount of TNF-α in *L. casei* + Strep group was similar to that from untreated animals. These results would indicate that the prevention of synovitis observed in *L. casei* + EC group is closely related to the absence of local TNF-α. 

It has been shown that TNF-α production and synovitis are related to the expression of IL-17 in joint draining lymph nodes [16,24]. With this in mind we analyzed the expression of IL-17 mRNA in popliteal and inguinal lymph nodes during *Salmonella* enterocolitis in animals with or without probiotic treatment. Figure 2B shows that the increase in IL-17 expression induced by *Salmonella* enterocolitis in lymph nodes draining the inflamed joints (EC group) was significantly lower (p<0.01) in animals fed with LCFM (*L. casei* + EC group). Expression of IL-17 mRNA was similar in *L. casei* + EC, Control and *L. casei* + Strep groups.

### Consumption of LCFM prevents *Salmonella*- induced enterocolitis

**Figure 3:**
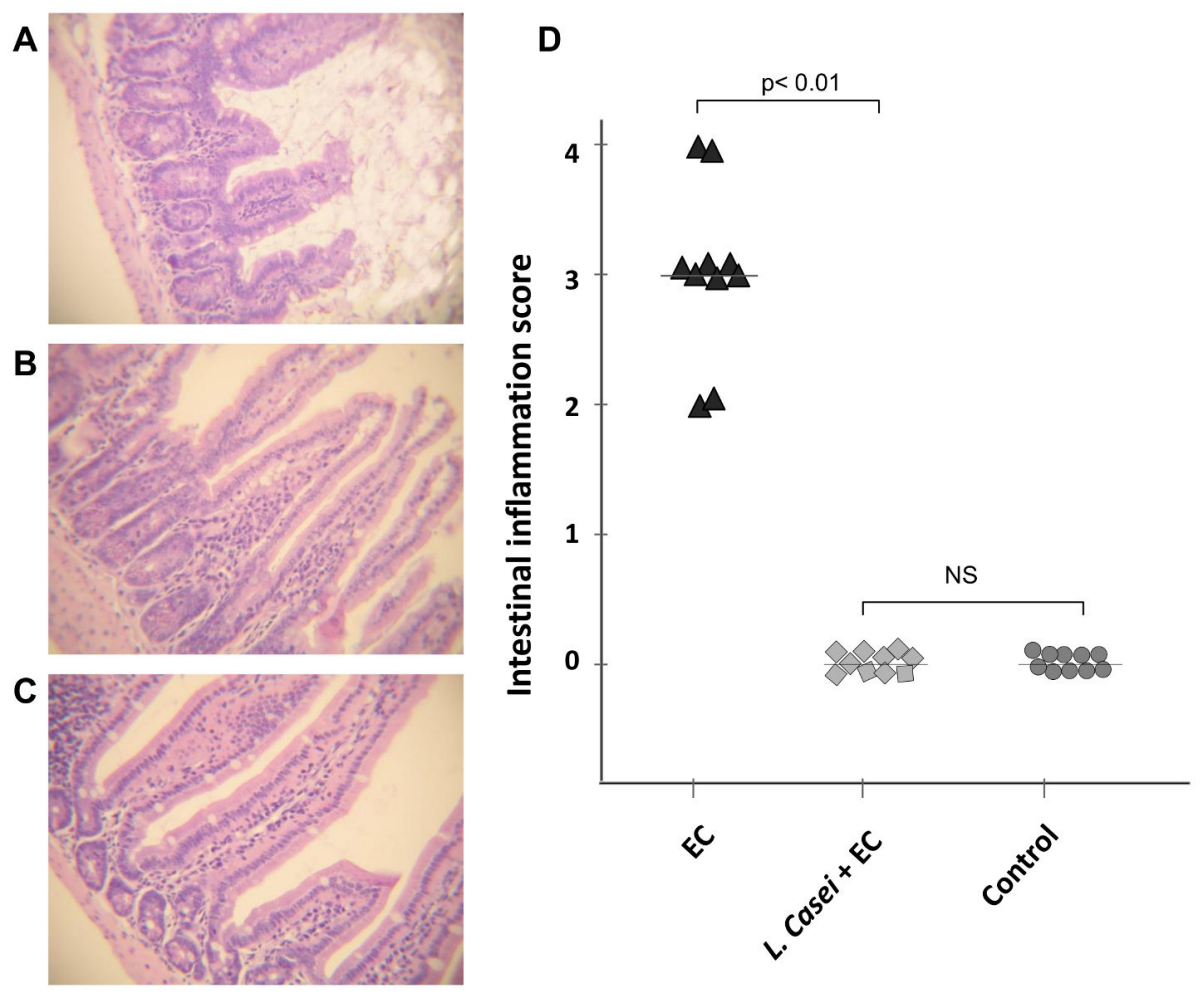
Effect of LCFM consumption on intestinal pathology during *Salmonella* enterocolitis. A to C: Histology of the intestine 2 days after oral infection with the pathogen. D: Intestinal inflammation scores. EC group: mice received 20 mg of streptomycin 24 h before intragastric infection with 3-4 x 10^3^ CFU of *S*. Enteritidis. *L. casei* + EC group: mice received the probiotic for a week before enterocolitis onset. Control group: untreated mice. (A) EC group: moderate enterocolitis, loss of normal villus architecture and height. (B) *L. casei* + EC group: regular display of intestinal villus, undistinguishable from Control group (C). HE; 40x. (NS): no significant differences. Data were collected from 3 independent experiments.

To this point our results show that ingestion of LCFM can prevent synovitis triggered by *S*. Enteritidis enterocolitis. The prominent link between intestinal and joint inflammation has been known for many years [25]. Therefore, it is likely that the beneficial effect of the probiotic on inflamed joints is related to the preservation of intestinal health during *Salmonella* enterocolitis. We next analyzed the effect of LCFM consumption on the intestinal inflammatory response to *S*. Enteritidis. Mice treated with streptomycin only (Strep group), fed with *L. casei* and streptomycin (*L. casei* + Strep) and animals without any treatment (Control group) were included as controls. Forty-eight hours after infection animals of the EC group presented signs of disease including diarrhea, rough hair coat, and lethargy. We found that *S*. Enteritidis induces diffuse enterocolitis, characterized by an epithelium diminished in height, mononuclear infiltration of the mucosa and submucosa, and loss of normal villus architecture (Figure 3A). Administration of LCFM for one week prior to infection prevented histological changes (*L. casei* + EC group; Figure 3B) as well as the clinical symptoms. The average histological score observed during *Salmonella* enterocolitis diminished from 3 in EC group to 0 in mice fed with the probiotic (*L. casei* + EC group); the latter was similar to Control group (Figure 3C and D). In addition, results obtained from *L. casei* + Strep group were similar to those presented by the Control group (data not shown).

The protective effect of LCFM observed on intestinal epithelium and clinical features could be related to the significant decrease in the bacterial invasion observed in the *L. casei* + EC group. It has been demonstrated that serovar Enteritidis has high blood invasiveness generating extra-intestinal focus of infection with elevated rates of mortality [26]. Our results clearly indicate that consumption of LCFM drastically diminishes *S*. Enteritidis invasiveness and shortens splenic persistence of the pathogen. Colonization and persistence of *S*. Enteritidis were analyzed at days 2, 5, and 14 postinfection (Table 3). Bacterial loads recovered at days 2 and 5 from Peyer’s patches were 10-fold lower in *L. casei* + EC group than in EC group. The effect of consumption of LCFM on spleen was even more dramatic. The intake of probiotics prior to *Salmonella* infection not only significantly decreased bacterial load, but also shortened infection period. *Salmonella* was still colonizing spleen at day 14 in EC group, whereas by that time this organ was sterile in animals from *L. casei* + EC group (Table 3). It is worth mentioning that in *L. casei* + EC group bacterial cultures were positive only after enrichment. 

**Table 3:** Effect of LCMF on colonization, persistence and survival during *Salmonella* enterocolitis.

**Treatment**	**Dose CFU/mice**	**Days p.i.**	**Peyer´s Patches (CFU/ml)^(a)^**	**Spleen (CFU/ml)^(a)^**	**Survival rate^(b)^**
EC	3-4 x 10^3^	2	52 (19-78)	65 (23-89)	5/5
		5	66 (24-113)	88 (32-111)	4/5
		14	Negative	55 (17-63)	4/5
*L. casei* + EC	3-4 x 10^3^	2	<5*	<5	5/5
		5	<5	<5	5/5
		14	Negative	Negative	5/5

Mice received 20 mg of streptomycin 24 h before intragastric infection with 3-4 x 10^3^ CFU of *Salmonella enterica* (EC group). *L. casei* + EC group received probiotic for a week before EC onset. At day 2, 5 and 14 after infection mice were sacrificed.

(a) Median (range). ^*^ *S. Enteritidis* was isolated only after enrichment in selenite broth. Survival rate: Number of survivors/total number of mice.

(b) Number of survivors/total number of mice.

Preserved intestinal epithelium and diminished bacterial invasiveness induced by LCFM correlated with 100% survival rate of animals from *L. casei* + EC group. Approximately 20% of mice with *Salmonella* enterocolitis die by day 7 post infection [16]; as shown in Table 3 no deaths occurred among mice fed with LCFM prior to infection. Neither live *Salmonella* nor their DNA were detected in joints or draining lymph nodes at any time tested for any group analyzed (data not shown). 

**Figure 4:**
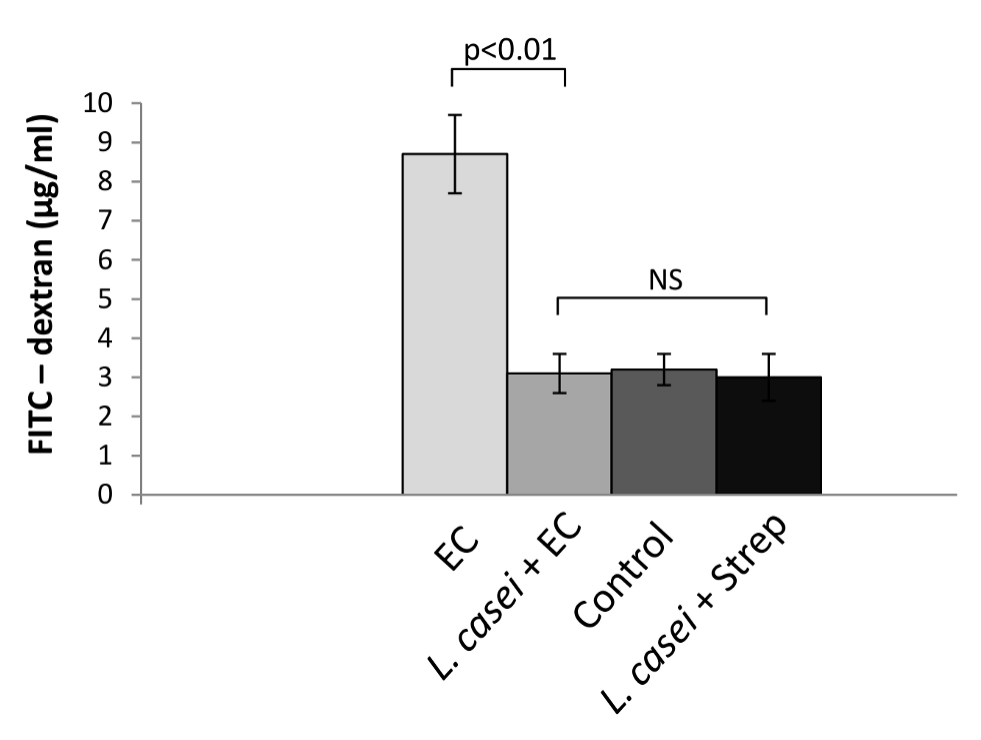
Effect of LCFM on intestinal permeability after *Salmonella* enterocolitis. A single dose of FITC–dextran was administered by gavage to the animals at day 2 post infection and the intensity of fluorescence was measured in serum 5 h later. EC group: mice received 20 mg of streptomycin 24 h before intragastric infection with 3-4 x 10^3^ CFU of *S*. Enteritidis. *L. casei* + EC group: mice received the probiotic for a week before enterocolitis onset. Control group: uninfected mice. *L. casei* + Strep group: animals were fed with the probiotic for a week and then received streptomycin. Results are expressed as mean +/- SD. Seven animals per group were analyzed. (NS): no significant differences. Representative data from 3 independent experiments.

It has been shown that intestinal permeability increases during enterocolitis [27]. We next decided to investigate the effect of LCFM on gut permeability during *Salmonella* enterocolitis. For that purpose, we administered a single dose of FITC–dextran by gavage to the animals at day 2 post infection and measured the intensity of fluorescence in serum 5 h later. As shown in Figure 4, the augment in gut permeability to macromolecules induced by *Salmonella* enterocolitis (EC group) was decreased by the consumption of LCFM to the same extent as found in healthy mice (*L. casei* + EC, *L. casei* + Strep and Control groups). It is likely then, that the enhancement of gut barrier function induced by LCFM consumption will prevent the intestinal response to the pathogen and consequently the associated joint sequelae. 

### Consumption of LCFM prevents the expression of local inflammatory molecules induced by *Salmonella* enterocolitis

**Figure 5:**
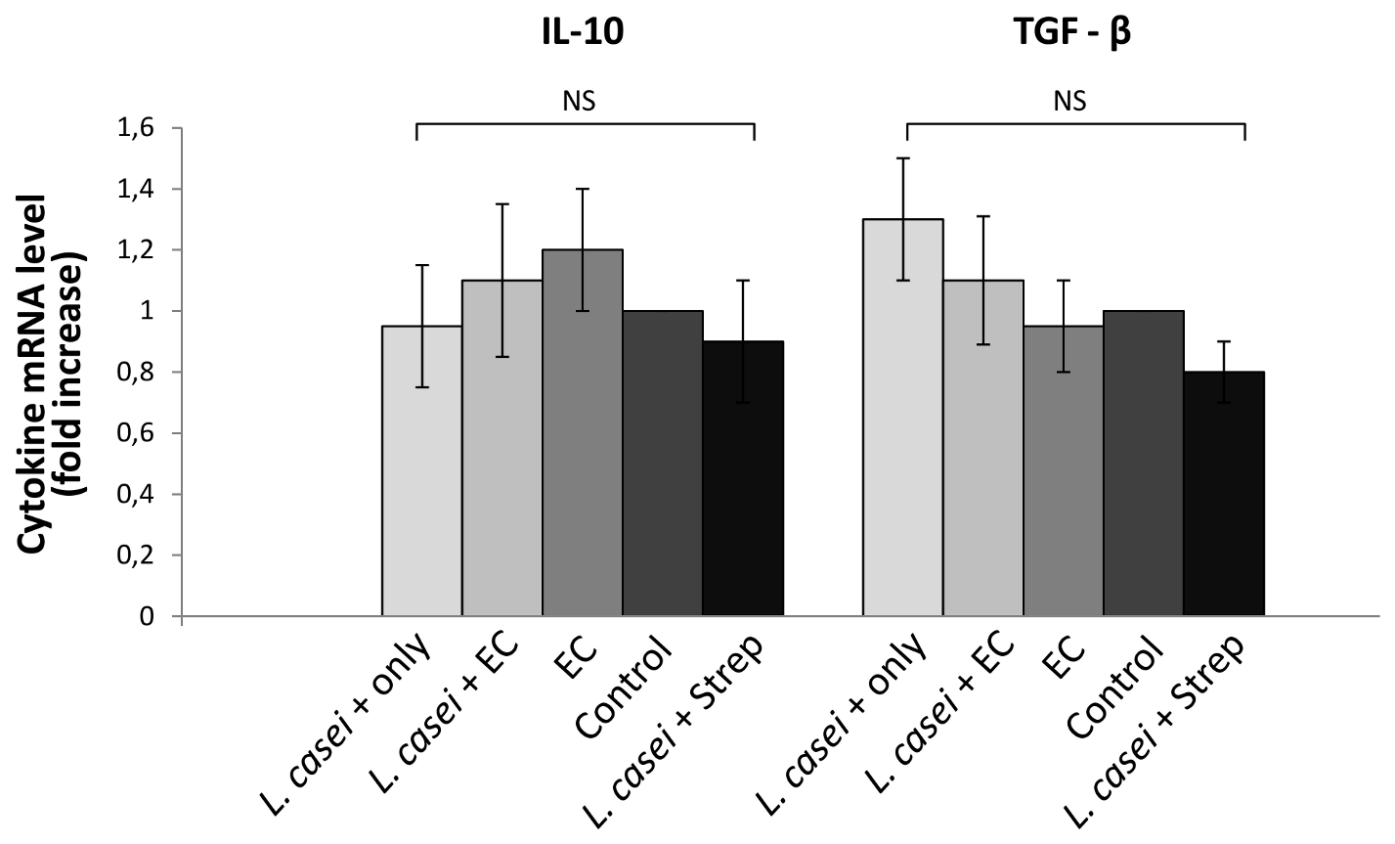
Effect of LCFM on intestinal IL-10 and TGF-β. Cytokines expression was analyzed by qPCR. *L. casei* only group: mice fed for a week with LCFM. EC group: mice received 20 mg of streptomycin 24 h before intragastric infection with 3-4 x 10^3^ CFU of *S*. Enteritidis. *L. casei* + EC group: mice received the probiotic for a week before enterocolitis onset. Control group: untreated mice. *L. casei* + Strep group: animals were fed with the probiotic for a week and then received streptomycin. Results are expressed as mean +/- SD. Four animals per group were analyzed. (NS): no significant differences. Representative data from 3 independent experiments.

Next we intended to analyze through which mechanism LCFM intake abrogates the inflammatory reaction of the gut against *S*. Enteritidis. Therapeutic efficacy of *L. casei* consumption is usually associated with the induction of anti-inflammatory molecules such as IL-10, TGF-β [28,29]. To test whether IL-10 and TGF-β are induced after LCFM consumption, we investigated the intestinal expression of these cytokines in mice treated with *L. casei* only. As shown in Figure 5 no increase in either cytokine expression was found in any experimental group studied. These results suggest that the mechanism through which LCFM prevents intestinal response to *S*. Enteritidis is not the elevation of the anti inflammatory cytokines.

**Figure 6:**
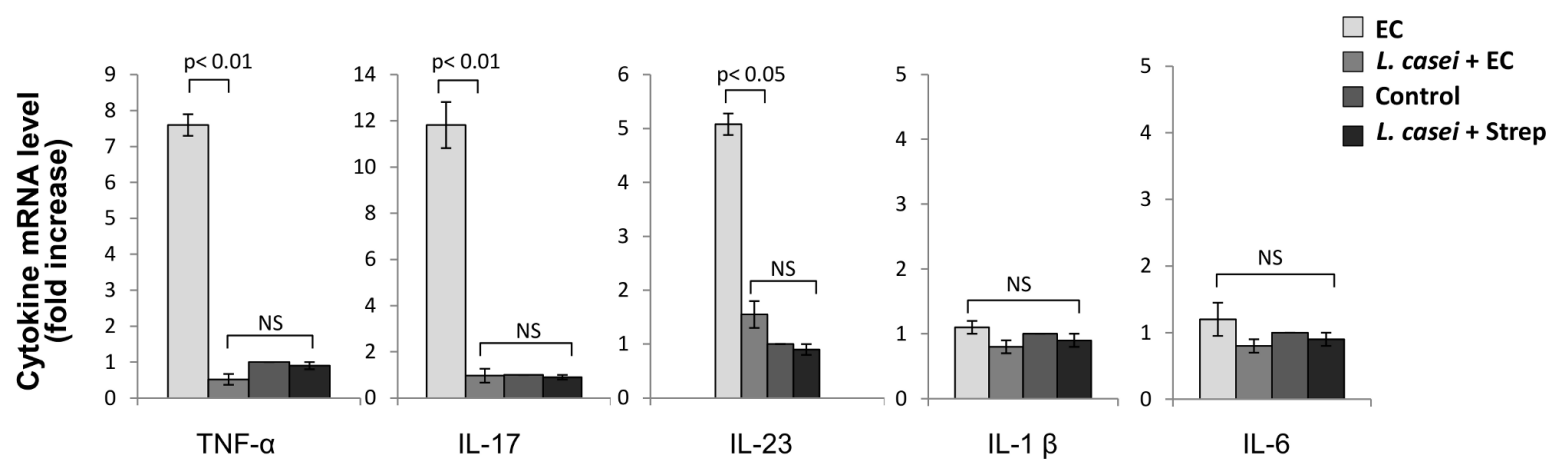
Effect of LCFM on intestinal cytokines after *Salmonella* enterocolitis. Cytokine expression was analyzed by qPCR 2 days after oral inoculation with 3-4 x 10^3^ UFC of the pathogen. EC group: mice received 20 mg of streptomycin 24 h before intragastric infection with 3-4 x 10^3^ CFU of *S*. Enteritidis. *L. casei* + EC group: mice received the probiotic for a week before enterocolitis onset. Control group: untreated mice. *L. casei* + Strep group: animals were fed with the probiotic for a week and then received streptomycin. Results are expressed as mean +/- SD. Seven animals per group were analyzed. (NS): no significant differences. Representative data from 3 independent experiments.

We then wondered whether probiotic treatment affects the expression of inflammatory cytokines triggered by *Salmonella* enterocolitis. Soon after intestinal infection *S. enterica* induces a significant increase in the expression of IL-17 and related cytokines such as TNF-α, IL-1β, IL-6 and IL-23 [5,16]. Interestingly, a substantial amount of data also links arthritis development to the expression of IL-17 and related cytokines [30]. As shown in Figure 6, during enterocolitis intestinal TNF-α is 7 times higher than in animals without *S*. Enteritidis infection (Control and *L. casei* + Strep groups); similarly, IL-17 and IL-23 are significantly elevated (11 and 5 times higher compared to controls, respectively). Interestingly, animals fed with the probiotic (*L. casei* + EC) did not respond to the pathogen burden, since the expression of IL-17 and related cytokines was similar to that seen in uninfected mice (Figure 6). No changes in the expression of intestinal IL-1β and IL-6 were observed at day 2 post infection (Figure 6).

**Figure 7:**
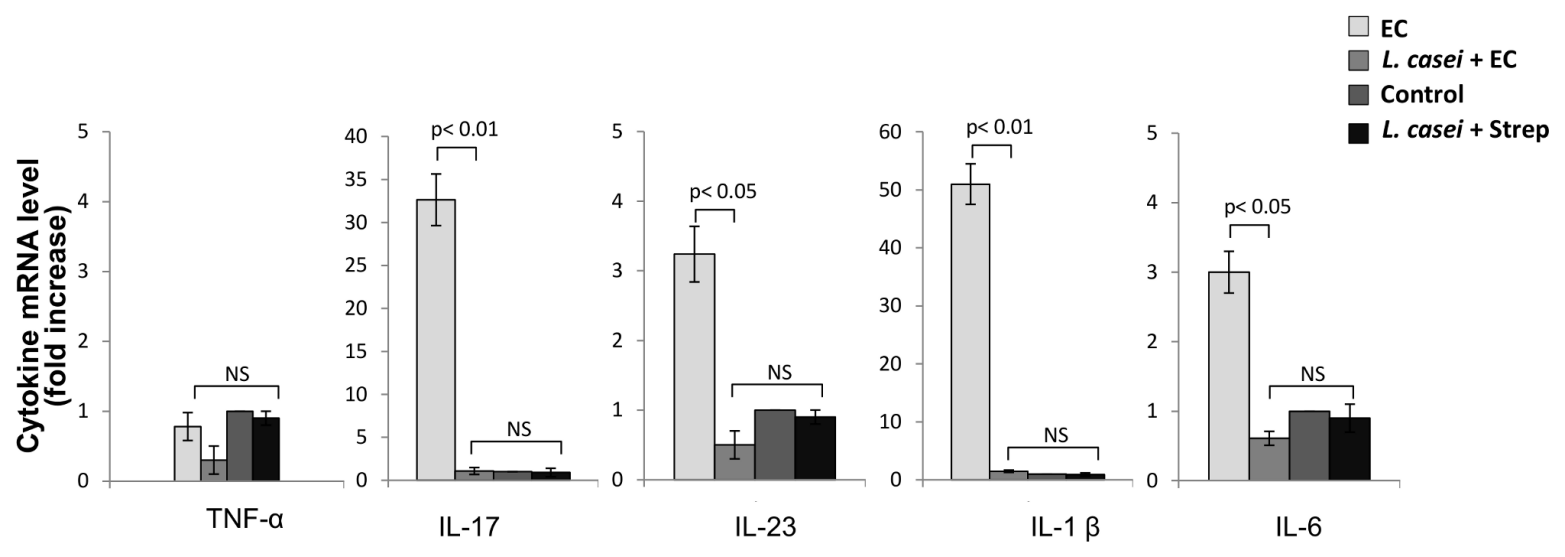
Effect of LCFM on mesenteric cytokines after *Salmonella* enterocolitis. Cytokine expression was analyzed by qPCR 5 days after oral inoculation with 3-4 x 10^3^ UFC of the pathogen. EC group: mice received 20 mg of streptomycin 24 h before intragastric infection with 3-4 x 10^3^ CFU of *S*. Enteritidis. *L. casei* + EC group: mice received the probiotic for a week before enterocolitis onset. Control group: untreated mice. *L. casei* + Strep group: animals were fed with the probiotic for a week and then received streptomycin. Results are expressed as mean +/- SD. Seven animals per group were analyzed. (NS): no significant differences. Representative data from 3 independent experiments.

The anti inflammatory effect of LCFM was even more dramatic in mesenteric lymph nodes. As shown in Figure 7, animals suffering from *Salmonella* enterocolitis (EC group) showed more than 30 fold increase in IL-17, 3-fold increase in IL-23, 50-fold increase in IL-1β and 3-fold increase in IL-6, compared to Control and *L. casei* + Strep groups. Consumption of LCFM (*L. casei* + EC group) abrogated the expression of all cytokines induced by *Salmonella* enterocolitis in mesenteric lymph nodes at day 5 post infection (Figure 7).

## Discussion

ReA is the development of sterile inflammatory arthritis as a sequel to a remote infection, often in the gastrointestinal tract. As stated before, in developing countries, more than thirty percent of ReA cases are associated with *Salmonella* enterocolitis [2]. Earlier we showed that oral infection with a low dose of *S*. Enteritidis induced to streptomycin-pretreated mice renders a suitable model for studying the pathogenesis of *Salmonella* ReA [16]. Here we demonstrate that consumption of LCFM prior to infection abolishes intestinal and joint inflammation triggered by *Salmonella* enterocolitis. 

Three main mechanisms of how probiotics can protect from intestinal inflammation have been described. First, probiotics may exclude or inhibit the growth of certain pathogens [31]; second, they may improve the gut barrier function [32]; and third, they can modulate mucosal and/or systemic immune response or metabolic functions [33]. The outcome of probiotic therapy also depends on the stage of the disease and the overall health status of the patient. 

We found that, at intestinal level, LCFM significantly reduced colonization and invasion of *S*. Enteritidis. Similar results have been recently reported by Castillo et al [34]; they found that continuous administration of *L. casei* to *Salmonella*-infected mice reduces significantly the pathogen counts in spleen, liver and large intestine. Adhesion of probiotic bacteria to the gastrointestinal surface is considered critical for the competitive exclusion of pathogens and for modulation of local and systemic immunological activities [35,36]. Specific adhesin-receptor interactions are one of the major mechanisms for the adhesion of bacteria to gastrointestinal surfaces. Thus, competition for adhesion between probiotics and intestinal pathogens could explain the beneficial effect of LCFM consumption on animals suffering from *Salmonella* enterocolitis. In support to this hypothesis, it has been shown that probiotics like *L. casei* Shirota present surface adhesins which are closely related to those found in *Salmonella* Typhimurium and Enteritidis [37]. In addition, it is likely that products secreted by *L. casei* and present in the fermented milk could interfere with *Salmonella* adhesion to intestinal epithelium. Fucose for instance, which is secreted by *L. casei* upon fermentation of N-acetylglucosamine, could certainly block *Salmonella Std* fimbriae. These adhesins, required for *Salmonella enterica* attachment and cecal colonization, recognize fucose residues on the epithelial cells [38–40].

We also found that *Salmonella* invasion is significantly diminished in LCFM-fed mice. This finding is in agreement with previous studies showing that probiotic consumption can strengthen the gut barrier function [41,42]. In mice suffering from dextran sulfate sodium-induced colitis *L. casei* leads to a significant protection against increased intestinal permeability [43]. Likewise, in our experiments, intake of LCFM prevented the increased permeability induced by *Salmonella* enterocolitis. Certain enteric pathogens regulate epithelial permeability by modifying expression and localization of tight junction proteins. *Salmonella* spp., for instance, disrupt tight junction proteins by means of effector proteins secreted through the type three secretion system (TTSS) [44,45]. Intimate attachment between bacteria and eukaryotic cells is an indispensable prerequisite condition to translocate effector proteins through TTSS-1 [46]. 

Therefore, prevention of *Salmonella* adhesion to the intestinal epithelium by LCFM could abrogate not only bacterial colonization but also translocation of effector proteins involved in disruption of intestinal barrier and bacterial invasion. Moreover, avoiding traslocation of effector proteins such as SopB, SopE and SopE2, could also explain the attenuation of the intestinal inflammatory response to *Salmonella* observed in LCFM fed mice. Different probiotic bacteria, including *L. casei*, can exert their anti inflammatory effect increasing the production of IL-10 and TGF-β by T cells [28,29]. In our model, however, neither intestinal IL-10 nor TGF-β expression was induced by LCFM. Our results are in line with a recent study in mice showing that *Bifidobacterium breve* -but not *L. casei*- is able to induce IL-10-producing T cells in the colon [47]. Furthermore, Chung et al showed that colonic TGF-β expression does not augment in *L. casei* treated animals [48]. It is likely then, that the anti inflammatory effect observed in the gut (and joints) of mice pre treated with the LCFM is related not to the induction of anti inflammatory cytokines but rather to the prevention of the expression of inflammatory molecules. 

In previous studies we showed that intestinal IL-17 induced by *Salmonella* enterocolitis is directly related to the induction of joint inflammation and that neutralization of IL-17 in *Salmonella* infected animals resulted in the abrogation of synovitis [16]. Furthermore, intestinal infection with Δ*invG* mutant of *Salmonella*, bearing a defective TTSS-1, triggers neither intestinal inflammatory IL-17 nor joint inflammation [16,49]. In the present work we show that consumption of LCFM prevents IL-17 intestinal response to *Salmonella* and at the same time blocks the induction of joint inflammation. 

Upon *Salmonella* infection, the IL-23/IL-17 axis is triggered in the intestinal mucosa. Macrophages and dendritic cells infected with *Salmonella* are a potential source of IL-23, a cytokine that helps to amplify the inflammatory response in intestinal tissue. Thus, IL-23 produced by phagocytes stimulates, in turn, T cells to secrete IL-17 [5]. Different T cells -like Th17, γδ and NKT- present in the intestinal mucosa and lamina propria express the receptor for IL-23 and constitute an important source of IL-17 during *Salmonella* infection [5,50]. Th17, γδ and NKT cells have been also implicated as a source of IL-17 production in different animal models of inflammation [51–56] and have been proposed as the link between gut inflammation and joint pathology in spondyloarthritis [57]. Trafficking of intestinal lymphocytes to the joints has been proposed as a theory to explain the concurrence of gut and joint inflammation [6,25,58]. On the other hand, differentiation of Th17 and γδ T cells requires a specific cytokine environment that includes IL-1β, IL-6 IL-23 and TGF-β [59–61]. Here, we show that the expression of these cytokines are induced during *Salmonella* enterocolitis and are abrogated by consumption of LCFM. In our model, the expression of intestinal inflammatory molecules concurs with the generation of joint lesions; in contrast, the abrogation of the expression of intestinal IL-17 and related cytokines prevents synovitis. This strongly suggests that the immunomodulatory effect of LCFM may have been achieved first in the gut and was subsequently expressed in a distant immune site. Our findings are in agreement with those reported by Inoue et al using a murine model of atopic dermatitis. They found that oral administration of *Lactobacillus* spp. diminished the expression of CD86 and IL-23 in Peyer´s patches and mesenteric lymph nodes as well as in skin lesions [62]. Our results suggest that LCFM consumption alters the intestinal milieu necessary for differentiation of the immune cells involved in the generation of joint inflammation. 

It is likely that down regulation of intestinal IL-23 by LCFM itself generates a protective effect on the joint. Numerous studies have suggested that overproduction of IL-23 is associated to joint pathologies [63–65] and the intestine appears as a key site of IL-23 production. As an example, it was found that in ankylosing spondylitis patients IL-23 expression is upregulated in the ileum. An explanation for this association has been recently achieved. Entheses contain resident lymphocytes expressing IL-23 receptor; IL-23 induces the expression of IL-17 and IL-22 in these T cells, hence triggering local joint inflammation [66]. Therefore, prevention of intestinal IL-23 expression during *Salmonella* enterocolitis could explain the abrogation of synovitis found in mice fed with LCFM.

Altogether our results show that consumption of LCFM prior to *Salmonella* enterocolitis onset abrogates intestinal inflammatory response to the pathogen. Expression of inflammatory cytokines was avoided in gut and joint tissues. Hence, prevention of the expression of these cytokines in mice fed with the probiotic could impair the differentiation of immune cells involved in the development of reactive arthritis. Furthermore, the abrogation of IL-23 expression in the intestine of mice receiving LCFM could account for the prevention of synovitis.

Regarding the pathogenesis of ReA, the role and mechanism of action of IL-23 are still not fully understood. However, it seems that IL-23/IL-17 is an important axis in ReA; thus interfering with the IL-23/IL-17 pathway could be a potential therapeutic target in the treatment of this disease. A deeper understanding of the functions and actions of IL-23 as well as the immune cells that generate it, may be useful in the management of new methods in the treatment of ReA, including the use of probiotics after the onset of arthritis.
